# Vitamin D Receptor Activator Use and Cause-specific Death among dialysis Patients: a Nationwide Cohort Study using Coarsened Exact Matching

**DOI:** 10.1038/srep41170

**Published:** 2017-01-31

**Authors:** Yoshitsugu Obi, Takayuki Hamano, Atsushi Wada, Yoshiharu Tsubakihara, The Committee of Renal Data Registry of the Japanese Society for Dialysis Therapy, Shigeru Nakai, Norio Hanafusa, Ikuto Masakane, Noritomo Itami, Kunihiro Yamagata, Toshio Shinoda, Junichiro James Kazama, Yuzo Watanabe, Takashi Shigematsu, Seiji Marubayashi, Osamu Morita, Naoki Kimata, Kenji Wakai, Satoshi Ogata, Kunitoshi Iseki, Keiichi Yamamoto, Ayumu Shintani

**Affiliations:** 1 Department of Nephrology, Osaka University Graduate School of Medicine, Suita, Osaka 565-0871, Japan; 2 Department of Comprehensive Kidney Disease Research, Osaka University Graduate School of Medicine, Suita, Osaka 565-0871, Japan; 3 Committee of Renal Data Registry of the Japanese Society for Dialysis Therapy, Bunkyo-ku, Tokyo 113-0033, Japan; 4 Department of Internal Medicine, Kita Saito Hospital, Asahikawa, Hokkaido 070-0030, Japan; 5 Jikei Institute Graduate School of Health Care Sciences, Osaka, Osaka 532-0003, Japan; 6 Department of Nephrology, Fujita Health University School of Medicine, Toyoake, Aichi 470-1192, Japan; 7 Department of Hemodialysis and Apheresis, the University of Tokyo Hospital, Tokyo 113-8655, Japan; 8 Kidney and Dialysis Center, Yabuki Hospital, Yamagata 990-0885, Japan; 9 Kidney Center and Department of Surgery, Nikko Memorial Hospital, Muroran 051-0005, Japan; 10 Department of Nephrology, University of Tsukuba, Tsukuba, Ibaraki 305-8575, Japan; 11 Dialysis Center, Kawakita General Hospital, Suginami-ku, Tokyo 166-8588, Japan; 12 Department of Nephrology, Hypertension, Diabetology, Endocrinology and Metabolism, Fukushima Medical University, Fukushima, Fukushima 960-1295, Japan; 13 Division of Internal Medicine, Kasugai Municipal Hospital, Kasugai, Aichi 486-0804, Japan; 14 Division of Nephrology and Blood Purification Medicine, Wakayama Medical University, Wakayama 641-8509, Japan; 15 Department of Artificial Organs, Tsuchiya General Hospital, Hiroshima, Hiroshima 730-8655, Japan; 16 Research Institute for Applied Mechanics, Kyushu University, Fukuoka, Fukuoka 816-8580, Japan; 17 Department of Blood Purification, Kidney Center, Tokyo Women’s Medical University, Shinjuku-ku, Tokyo 162-8666, Japan; 18 Department of Preventive Medicine, Nagoya University Graduate School of Medicine, Nagoya, Aichi 466-8550, Japan; 19 Faculty of Health Sciences, Hiroshima International University, Higashi-hiroshima, Hiroshima 739-2695, Japan; 20 Clinical Research Support Center, Tomishiro Central Hospital, Tomishiro, Okinawa 901-0243, Japan; 21 Department of Medical Innovation, Osaka University Hospital, Suita, Osaka 565-0871, Japan; 22 Department of Clinical Epidemiology and Biostatistics, Osaka University Graduate School of Medicine, Suita, Osaka 565-0871, Japan

## Abstract

Vitamin D receptor activators (VDRA) may exert pleiotropic effects on cardiovascular disease, malignancy, and infections among dialysis patients, but recent studies have mainly focused on cardiovascular outcomes. Among 8,675 patients who started dialysis in 2007 and who survived until January 1, 2010, listed in the Renal Data Registry of the Japanese Society for Dialysis Therapy, 5,365 VDRA users were matched to 3,203 non-users based on clinically relevant variables at the end of 2009 using the coarsened exact matching procedure. Until December 31, 2011, a total of 1,128 deaths occurred, of which 468 (42%) were cardiovascular deaths, 229 (20%) were infection-related deaths, and 141 (12%) were malignancy-related deaths. Multivariable survival analyses accounting for intra-region correlation revealed that VDRA use was significantly associated with lower rates of infection- and malignancy-related deaths [subhazard ratio 0.62 (95% CI, 0.52–0.73) and 0.70 (95% CI, 0.50–0.97), respectively] but not with cardiovascular death [subhazard ratio 0.86 (95% CI, 0.72–1.04)]. Future randomized clinical trials with a sufficient sample size and an adequate follow-up period are warranted to test the clinical effectiveness of VDRA on infection and malignancy, rather than cardiovascular disease, among dialysis patients.

Vitamin D may exert protective effects against cardiovascular disease, infection, and malignancy^1^, which are among the most frequent causes of death in patients with end-stage renal disease (ESRD)^2^,^3^. Given the high prevalence of vitamin D deficiency and the diminished activity of renal 1α-hydroxylase in ESRD patients, treatment with vitamin D receptor activators (VDRAs) may offer a survival benefit, in addition to their effects on mineral and bone metabolism, by reducing the risk of these disease conditions^4^.

There are conflicting data on the association of VDRA use with mortality. Some studies support the survival benefit of VDRA use in patients with chronic kidney disease (CKD), including ESRD^5^,^6^,^7^,^8^, while another study found no association with mortality using an instrumental variable approach that may reduce the influence of unmeasured confounders^9^. Moreover, recent clinical trials have shown no benefit of VDRA use in terms of preventing the progression of left ventricular hypertrophy among patients with CKD^10^,^11^. Instrumental variable method, however, also has several limitations that may bias the results toward null^12^,^13^. Additionally, VDRA, which downregulates renin expression, may not be effective in preventing cardiovascular disease because in the current clinical practice, many patients with CKD receive renin-angiotensin system inhibitors^10^,^11^. Therefore, we used a nationwide cohort of Japanese dialysis patients to test our hypothesis that VDRA use is more strongly associated with infection- or malignancy-related death compared to cardiovascular death.

## Results

**Figure 1:**
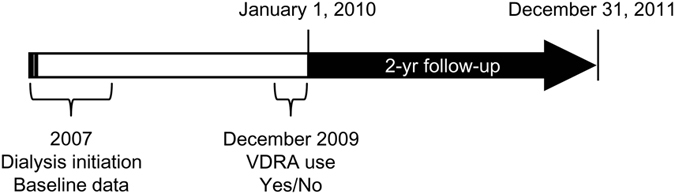
Study design. Among dialysis patients who survived until January 1, 2010, VDRA users and non-users were defined based on data on VDRA use extracted from the 2009 survey and matched based on demographics, comorbidities, and laboratory variables at dialysis initiation in 2007.

Among 8,675 ESRD patients who started dialysis in 2007 and who survived until January 1^st^, 2010, a total of 5,365 VDRA users as of December 2009 were matched to 3,203 non-users based on age, sex, diabetes, history of CVD, initial dialysis modality (HD or PD), and estimated GFR at dialysis initiation using the coarsened exact matching procedure (Fig. 1)^14^. Given the low prevalence of VDRA use during the pre-dialysis period in Japan (i.e. <9%)^15^, we assumed that VDRA use reflects the degree of exposure to VDRA after dialysis initiation and that clinical data at dialysis initiation can be used as pretreatment variables for causal inference. Among 5,365 VDRA users, 3,782 (71%) and 1,746 (34%) patients used intravenous VDRAs (i.e., calcitriol or maxacalcitol) and oral VDRAs (i.e., calcitriol, alfacalcidol, or falecalcitriol), respectively. At dialysis initiation, patients in the matched cohort were 65 ± 13 years old and had a mean GFR of 4.7 ± 2.3 mL/min/1.73 m^2^ at dialysis initiation; 65% were male, 42% were diabetic, 20% had history of cardiovascular disease, and 4% received peritoneal dialysis (Table 1). Compared to non-VDRA users, VDRA users showed small differences in serum albumin concentrations (3.4 ± 0.6 mg/dL vs. 3.3 ± 0.6 mg/dL; standardized difference = 0.16) and central venous catheter use as vascular access (28% vs. 34%; standardized difference = 0.11). There were no meaningful differences (i.e., standardized difference < 0.1) in the other variables, including hemoglobin and serum concentrations of corrected calcium, phosphorus, and C-reactive protein.

As of the end of 2009, there were no meaningful between-group differences in the prevalence of calcium carbonate use (60% vs. 61%), cinacalcet use (3.4% vs. 4.7%), or prior parathyroid interventions, including parathyroidectomy and percutaneous ethanol injection (0.5% vs. 0.6%), while a small difference favoring VDRA users was observed in non-calcium containing phosphorus binder use (32% vs. 26% in non-VDRA users; standardized difference = 0.11).

**Figure 2:**
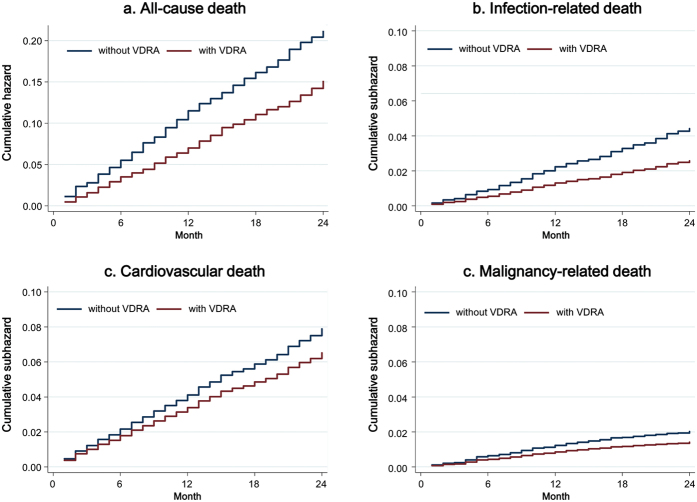
Cumulative (sub)hazards for (**a**) all-cause, (**b**) cardiovascular, (**c**) infection-related, and (**d**) malignancy-related death in the matched cohort of 8,568 patients including 3,203 VDRA users and 5,365 non-VDRA users as of the end of 2009. Abbreviation: VDRA, vitamin D receptor activator.

**Figure 3:**
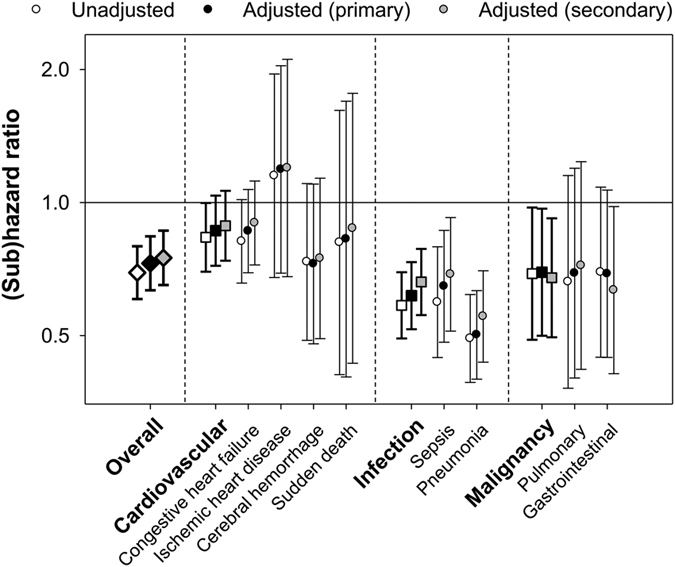
Hazard/subhazard ratio of vitamin D activator use for all-cause, cardiovascular, infection-related, and malignancy-related death. The primary adjusted model included central venous catheter use, body weight, mean arterial blood pressure, hemoglobin, and serum concentrations of albumin, calcium, phosphorus, and C-reactive protein. The secondary adjusted model included covariates in the primary model plus the use of calcium carbonate, non-calcium containing phosphorus binders (i.e., sevelamer hydrochloride and lanthanum carbonate), and cinacalcet, and history of invasive parathyroid interventions (i.e., parathyroidectomy and percutaneous ethanol injection therapy) as of the end of 2009.

During the follow-up between January 1^st^, 2010 and December 31^st^, 2012 [median 24 (IQR, 24 to 24) months], 1,128 deaths occurred, of which 468 (42%) were cardiovascular deaths, 229 (20%) were infectious deaths, and 141 (12%) were due to malignancy. All-cause mortality was 9.3 (95% CI, 8.8–9.8) per 100 patient-years, and the incidence rates for cardiovascular, infection-related, and malignancy-related death were 4.0 (95% CI, 3.7–4.3), 1.9 (95% CI, 1.7–2.1), and 0.9 (95% CI, 0.8–1.1) per 100 patient-years. VDRA users appeared to have lower mortality irrespective of cause of death (Fig. 2). Primary multivariable Cox regression analyses accounting for intra-region correlation showed that VDRA use was significantly associated with a lower risk of all-cause death [hazard ratio 0.73 (95% CI, 0.63–0.84)]. Likewise, in competing-risks regression analyses, a reduced risk associated with VDRA use was observed for infection- and malignancy-related death [subhazard ratios 0.62 (95% CI, 0.52–0.73) and 0.70 (95% CI, 0.50–0.97), respectively] but not with cardiovascular death [subhazard ratio 0.86 (95% CI, 0.72–1.04)] (Fig. 3). Additionally, VDRA use showed consistent associations with subcategories of cardiovascular and infection-related death; VDRA use was associated with lower mortality due to sepsis and pneumonia, but not with congestive heart failure, ischemic heart disease, cerebral hemorrhage, or sudden death. The associations of VDRA use with death due to pulmonary and gastrointestinal cancer did not reach statistical significance, likely due to the limited number of each outcome. Sensitivity analyses with further adjustment for therapies that may be on the causal pathway between VDRA use and mortality (i.e., the use of calcium carbonate, non-calcium phosphorus binders, and cinacalcet and prior invasive parathyroid interventions as of the end of 2009) slightly attenuated these associations in most outcomes but did not change their significance.

## Discussion

In this nationwide cohort of Japanese dialysis patients, we demonstrated the difference in the strength of the relationship between VDRA use and three major causes of death; VDRA use was associated with lower infection- and malignancy-related mortality but not with cardiovascular mortality.

Vitamin D stimulates innate immunity and enhances antimicrobial activity by upregulating the synthesis of the antimicrobial peptide cathelicidin^16^, which may explain the association between VDRA and infection-related death observed in this study. Interestingly, the association with lower mortality due to pneumonia is consistent with previous studies demonstrating a lower incidence of hospitalization due to acute respiratory infection among hemodialysis patients treated with vs. without oral VDRAs^17^,^18^. Another study also found that both intravenous and oral VDRAs showed an association with lower infection-related mortality^19^. Our study had relatively greater study power due to a larger sample size and thus enabled the examination of subcategory outcomes (i.e., sepsis and pneumonia) independently. The lower risk associated with VDRA use was consistently observed for both causes of death, suggesting a robust relationship between VDRA use and infection-related death.

Given that vitamin D also has antiproliferative effects, activates apoptotic pathways, inhibits angiogenesis, and promotes cell differentiation, nutritional vitamin D supplementation or VDRA use may prevent the development and progression of various cancers^20^. Although, there are scarce data on the benefit of VDRA use on cancer mortality among hemodialysis patients, partly due to its relatively low incidence of malignancy-related death compared to cardiovascular death, pre-transplant vitamin D deficiency is associated with a risk of post-transplant malignancy among kidney transplant recipients, who have decreased kidney function and high incidence of cancer^21^. Moreover, kidney transplant recipients with VDRAs, compared to those without VDRAs, showed a lower incidence of cancer^22^, which is consistent with the results of the present study.

However, VDRA may be less effective in reducing cardiovascular risk for patients receiving renin-angiotensin system (RAS) inhibitors. VDRAs have been suggested to have a protective effect against cardiovascular disease because vitamin D suppresses RAS activity by downregulating renin expression in the kidney via its interaction with the vitamin D receptor^23^. Indeed, a small prospective observational study, which enrolled hemodialysis patients in 1992 when RAS inhibitors were not frequently used in this population, showed that patients on oral VDRA treatment exhibited lowered cardiovascular mortality compared to non-oral VDRA users^24^. However, recent clinical trials failed to demonstrate the cardio-protective effect of VDRA among pre-dialysis CKD patients with high prevalence (i.e., 70–80%) of RAS inhibitor use^10^,^11^. The prevalence of RAS inhibitor use is also increasing in the hemodialysis population from ~30% in 2002 to ~40% in 2008 in Japan^25^,^26^. These secular trends may explain the discrepancy among studies and the reason why the present study showed a trend, albeit not statistically significant due to the limited number of events, toward lower cardiovascular mortality among dialysis patients on VDRAs.

Although other mineral and bone disorder-related therapies, such as cinacalcet, invasive parathyroid interventions (i.e., parathyroidectomy and percutaneous ethanol injection therapy), and non-calcium containing phosphorus binders may partly explain the association between VDRA use and mortality^27^,^28^,^29^,^30^, the between-group differences in their prevalence as of the end of 2009 were not meaningful, and the adjustment for these potential intermediates slightly attenuated but did not change the significance in the associations of VDRA use with all-cause and cause-specific mortality. Importantly, the prevalence of cinacalcet use was low (i.e., 4% overall) because cinacalcet became available in clinical practice from January 2008 in Japan. Thus, this study did not enable an evaluation of the impact of cinacalcet on the association between VDRA use and mortality as suggested in the ADVANCE study^31^.

There are two studies showing conflicting results on the VDRA-mortality association. Naves-Diaz *et al*. showed that in a propensity-matched cohort of incident hemodialysis patients, oral VDRA use was associated with a similar reduction (i.e., 40–50%) in the risk of mortality across causes of death, including cardiovascular disease, infection, and malignancy^5^. The reason for the difference in the results between their study and the present study is unclear, but may be due to the difference in the definition of VDRA use and patient characteristics (i.e., race, history of cardiovascular disease, and the use of CV catheter, RAS inhibitor, and vitamin D supplementation). In contrast, Tentori *et al*. showed no association between VDRA use and all-cause mortality using the instrumental variable method, an alternative approach for estimating the causal associations using observational data. It uses an exogenous variable that is associated with exposure but not with outcome, and this method is considered less biased because it can eliminate both residual and unmeasured confounding under ideal conditions. However, it is challenging to identify appropriate variables that satisfy the assumptions of instruments. Furthermore, the instrumental variable method relies on only a portion of the total variance and leads to less precise and more conservative estimates than regression models, as indicated by wide standard errors, especially in cases with weak instrument relevance^12^. Treatment preferences of groups (e.g., clinical facilities) have been proposed as instruments, but several guidelines on VDRA might have made the preference less relevant to this exposure^32^,^33^. Also, this approach is valid only when unmeasured confounding effects exist within groups but not between groups, which is likely not the case with the real world scenario^13^. These limitations might have biased the results toward the null in the previous study of VDRA among hemodialysis patients using the instrumental variable method^9^.

We also acknowledge several imitations in this study. First, the observational nature of this study does not allow us to confirm causality between VDRA use and cause-specific death, and there may be residual and unmeasured confounders. Specifically, neither serum 25-hydroxyvitamin D nor 1,25-dihydroxyvitamin D was available in this study, which was based on nation-wide surveys consisting of practical questionnaires, because their measurements have not been reimbursed and not been routinely performed under the bundled payment system in Japan. Second, we used the baseline Cox models and competing-risks regression models with VDRA use as of December 2009 as the exposure variable but did not take into account the doses or their changes overtime. We also do not have exact data on VDRA usage at dialysis initiation, although the prevalence of VDRA use during the pre-dialysis period was reasonably low in Japan (i.e. <9%)^15^. Third, there may be survivor bias because our cohort consisted of dialysis patients who initiated dialysis in 2007 and survived until January 1^st^, 2010, which might have resulted in bias toward the null, particularly for cardiovascular mortality, by excluding fragile patients. Finally, our result may not be extrapolated to patients with pre-dialysis CKD or those receiving dialysis treatments in countries with different proportions of causes of death or a different prescription pattern of RAS inhibitors. The proportion of patients on peritoneal dialysis was also limited.

In conclusion, VDRA use was associated with infection- and malignancy-related mortality, but not with cardiovascular mortality, among dialysis patients. Future randomized clinical trials with a sufficient sample size and an adequate follow-up period are needed to test the clinical effectiveness of VDRA on infection and malignancy, rather than cardiovascular disease, in this population.

## Methods

The Japanese Society for Dialysis Therapy conducts annual surveys of dialysis facilities throughout Japan in December. The surveys address epidemiological background, treatment conditions and the outcomes of treatment with dialysis. Dataset JRDR-13104 was used with the permission of the Committee of the Renal Data Registry of the Japanese Society for Dialysis Therapy (JRDR). The study protocol was approved by the Medicine Ethics Committee of the Japanese Society for Dialysis Therapy as exempt from informed consent. The study was performed in accordance with the Declaration of Helsinki.

### Study Cohort Restriction

**Figure 4:**
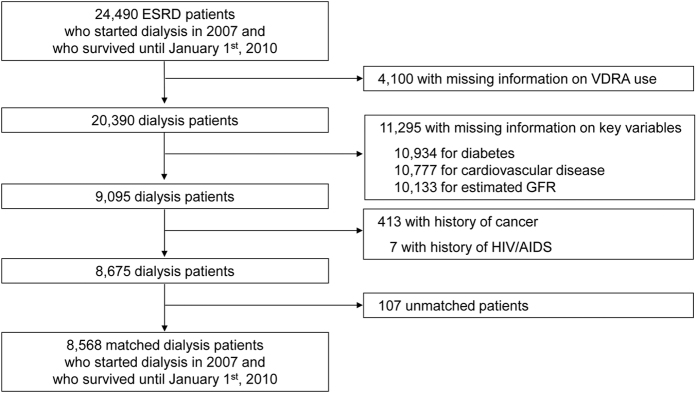
Study flow diagram. Abbreviations: ESRD, end-stage renal disease; VDRA, vitamin D receptor activator; GFR, glomerular filtration rate; AIDS, acquired immunodeficiency syndrome; HIV, human immunodeficiency virus.

To examine the association of exposure to VDRA with all-cause and cause-specific mortality, we identified 24,490 ESRD patients who started dialysis treatment during 2007 and who survived until January 1^st^, 2010 (Fig. 4). Information on dialysis patients and data on VDRA use were available in the surveys at 2007 and 2009, respectively. Thus, data on demographics, comorbidities, and laboratory variables at dialysis initiation were extracted from the 2007 survey. Hemoglobin and serum concentrations of albumin, creatinine, calcium, phosphorus, and C-reactive protein were measured using standard laboratory techniques at each center. VDRA users and non-users were defined based on data on VDRA use extracted from the 2009 survey. VDRA available in Japan between 2007 and 2009 included oral calcitriol, oral alfacalcidol, oral falecalcitriol, intravenous calcitriol, and intravenous maxacalcitol. Out of 24,490 patients, we excluded 4,100 with missing data on VDRA use and 11,295 with missing information on diabetes, history of cardiovascular disease, or estimated glomerular filtration rate (GFR). We also excluded 413 and 7 patients with history of cancer and history of human immunodeficiency virus infection and acquired immune deficiency syndrome (HIV/AIDS), respectively.

Among the remaining 8,675 dialysis patients, 5,365 VDRA users were matched to 3,203 non-users based on age, sex, diabetes, history of CVD, initial dialysis modality (HD or PD), and estimated GFR at dialysis initiation using the coarsened exact matching procedure^14^. First, we coarsened age and estimated GFR at dialysis initiation into categories using cut-points of 50, 60, 70, and 80 years and Sturge’s rule, respectively. We then sorted all patients by each stratum of the coarsened variables, as well as by sex, diabetes, central venous catheter as vascular access, and dialysis modality (PD or HD). Within each stratum that included at least one patient in each group, non-VDRA users were given a weight of 1, and VDRA users were given a weight that equalized the ratio of sum of weights in each group of the stratum to the ratio of total matched patients on each group. Coarsened exact matching has several advantages over other matching approaches, including propensity-score matching; requiring fewer ad-hoc post-estimation assumptions about how to define a match; automatic balancing of treatment and control populations; and superior computational properties for large datasets. It is also particularly suitable for applications where most independent variables can be categorized appropriately. There were no meaningful differences in variables in Table 1 except for a small difference in estimated GFR among included vs. excluded patients (5.0 ± 2.4 vs. 5.7 ± 4.6 mL/min/1.73 m^2^, Supplemental Table 1).

### Statistics

Data are presented as the mean ± SD or percentage, as appropriate. Differences between groups were compared by standardized differences due to the large sample size of this study^34^,^35^. Standardized differences of 0.8, 0.5, and 0.2 in absolute values are considered large, medium, and small differences, and ≥0.1 was defined as meaningful imbalance^34^,^35^. The primary outcome was time to infection-related death. The secondary outcomes included time to all-cause, cardiovascular, and malignancy-related death. Information on all-cause, cardiovascular, infection-related, and malignancy-related death were extracted from the surveys at 2010 and 2011. Cardiovascular death was defined as death due to heart failure, pulmonary edema, ischemic heart disease, arrhythmia, endocarditis, valvular disease, other cardiac disease, subarachnoid hemorrhage, cerebral hemorrhage, cerebral infarction, other cerebrovascular disease, or sudden death. Infection-related death was defined as death due to sepsis, pneumonia, gastrointestinal infection, cholecystitis/cholangitis, peritonitis, tuberculosis, HIV/AIDS, hepatitis virus infection, influenza virus infection, or other infection. The association of VDRA use and cause-specific mortality was examined by competing-risks regression based on Fine and Gray’s proportional subhazards model^36^ with and without adjustment for central venous catheter use, body weight, mean arterial blood pressure, hemoglobin, and serum concentrations of albumin, calcium, phosphorus, and C-reactive protein, using the other causes of death as competing risks. With regard to the sensitivity analyses, we further adjusted for other mineral and bone disorder-related therapies, including calcium carbonate, non-calcium containing phosphorus binders (i.e., sevelamer hydrochloride and lanthanum carbonate), cinacalcet, and history of invasive parathyroid interventions (i.e., parathyroidectomy and percutaneous ethanol injection therapy) as of the end of 2009. Clustered sandwich estimator was used to allow intragroup correlation within regions (i.e., prefectures). The multiple imputation method with 5 data sets was used to address missing values using multivariate normal regression based on all baseline data and an indicator of death. We performed Cox regression analyses to determine the association of VDRA use and all-cause mortality. *P*-values < 0.05 were considered significant.

## Additional Information

**How to cite this article**: Obi, Y. *et al*. Vitamin D Receptor Activator Use and Cause-specific Death among dialysis Patients: a Nationwide Cohort Study using Coarsened Exact Matching. *Sci. Rep.* **7**, 41170; doi: 10.1038/srep41170 (2017).

**Publisher's note:** Springer Nature remains neutral with regard to jurisdictional claims in published maps and institutional affiliations.

## Supplementary Material

Supplemental Table 1

## Figures and Tables

**Table 1:** Characteristics at dialysis initiation in 2007 between VDRA users vs. non-users as of December 2009.

Variables	Missing	VDRA use as of December 2009		
		Non-users n = 3,203 (37%)	Users n = 5,365 (63%)	Standardized difference
Age (years)	(0%)	65 ± 13	65 ± 13	0.00
Male (%)	(0%)	65%	65%	0.00
Diabetes (%)	(0%)	42%	42%	0.00
History of cardiovascular disease (%)	(0%)	20%	20%	0.00
Estimated GFR (mL/min/1.73 m^2^)	(0%)	4.9 ± 2.3	4.9 ± 2.3	0.01
Peritoneal dialysis (%)	(0%)	4%	4%	0.00
Central venous catheter use (%)	(1%)	34%	28%	0.11
Body weight (kg)	(6%)	59 ± 13	60 ± 13	0.03
Mean atrial blood pressure (mmHg)	(7%)	105 ± 18	105 ± 17	−0.01
*Laboratories*				
Albumin (g/dL)	(7%)	3.3 ± 0.6	3.4 ± 0.6	0.16
Hemoglobin (g/dL)	(1%)	8.3 ± 1.5	8.4 ± 1.6	0.06
Corrected calcium (mg/dL)	(5%)	7.8 ± 1.1	7.8 ± 1.1	−0.04
Phosphorus (mg/dL)	(5%)	6.1 ± 1.9	5.9 ± 1.8	−0.09
C-reactive protein (mg/dL)	(19%)	1.7 ± 4.2	1.5 ± 3.7	−0.07

Note: Values are expressed as the mean ± SD or percentage as appropriate. SI conversion factors: to convert hemoglobin to g/L, multiply by 10; albumin to g/L, multiply by 10; calcium to mmol/L, multiply by 0.25; phosphorus to mmol/L, multiply by 0.323; C-reactive protein to nmol/L, multiply by 95.24. Abbreviations: GFR, glomerular filtration rate. Standardized differences of 0.8, 0.5, and 0.2 in absolute values are considered large, medium, and small differences, respectively, and ≥0.1 was defined as a meaningful imbalance^34^,^35^.
