# Supplementary material for: Vitamin D Receptor Activator Use and Cause-specific Death among dialysis Patients: a Nationwide Cohort Study using Coarsened Exact Matching
Source: Sci Rep. 2017 Jan 31;7:41170. doi: 10.1038/srep41170 (PMC5282519; doi:10.1038/srep41170)
Supplement: Supplemental Table 1 [file srep41170-s1.pdf]

Supplementary Material

Vitamin D Receptor Activator Use and Cause-specific Death among dialysis Patients: a Nationwide Cohort Study using Coarsened Exact Matching

Yoshitsugu Obi, MD, PhD; Takayuki Hamano, MD, PhD; Atsushi Wada, MD, PhD; Yoshiharu Tsubakihara, MD, PhD; and the Committee of Renal Data Registry of the Japanese Society for Dialysis Therapy.

Supplemental Table 1. Characteristics at dialysis initiation in 2007 between included vs. excluded patients in this study.

Variables	Excluded n=15,921 (65%)	Missing	Included n=8,568 (35%)	Missing	Standardized difference
Age (years)	65 ± 13	1 (0%)	65 ± 13	0 (0%)	0.02
Male	65%	0 (0%)	64%	0 (0%)	0.02
Diabetes	47%	13,832 (87%)	45%	0 (0%)	0.04
CVD history	23%	13,639 (86%)	21%	0 (0%)	0.04
Estimated GFR (mL/min/1.73m2)	5.7 ± 4.6	12,791 (80%)	5.0 ± 2.4	0 (0%)	-0.19
Peritoneal dialysis	6%	0 (0%)	4%	0 (0%)	0.04
Central venous catheter use	26%	12,027 (76%)	30%	68 (1%)	-0.09
Body weight (kg)	59 ± 14	13,067 (82%)	59 ± 13	519 (6%)	0.02
Mean atrial blood pressure (mmHg)	104 ± 18	13,167 (83%)	105 ± 17	624 (7%)	0.02
Laboratories					
Albumin (g/dL)	3.3 ± 0.6	13,025 (82%)	3.3 ± 0.6	594 (7%)	0.05
Hemoglobin (g/dL)	8.4 ± 1.6	12,807 (80%)	8.3 ± 1.6	77 (1%)	-0.03
Corrected calcium (mg/dL)	7.8 ± 1.1	12,932 (81%)	7.8 ± 1.1	412 (5%)	0.00
Phosphorus (mg/dL)	5.8 ± 1.9	13,005 (82%)	6.0 ± 1.8	428 (5%)	0.07
C-reactive protein (mg/dL)	1.7 ± 4.0	13,360 (84%)	1.6 ± 3.8	1,676 (20%)	-0.04
Vitamin D receptor activator use	61%	4,100 (26%)	63%	0 (0%)	-0.04

Note: Values are expressed as mean±SD or percentage, appropriately. SI conversion factors: To convert hemoglobin to g/L, multiply by 10; albumin to g/L, multiply by 10; calcium to mmol/L, multiply by 0.25; phosphorus to mmol/L, multiply by 0.323; C-reactive protein to nmol/L, multiply by 95.24. Abbreviations: GFR, glomerular filtration rate. Standardized differences of 0.8, 0.5, and 0.2 in absolute values are considered large, medium, and small differences.
